# Using Machine Learning to Predict the Duration of Atrial Fibrillation: Model Development and Validation

**DOI:** 10.2196/63795

**Published:** 2024-11-22

**Authors:** Satoshi Shimoo, Keitaro Senoo, Taku Okawa, Kohei Kawai, Masahiro Makino, Jun Munakata, Nobunari Tomura, Hibiki Iwakoshi, Tetsuro Nishimura, Hirokazu Shiraishi, Keiji Inoue, Satoaki Matoba

**Affiliations:** 1 Department of Cardiac Arrhythmia Research and Innovation Graduate School of Medical Science Kyoto Prefectural University of Medicine Kyoto Japan; 2 Department of Cardiovascular Medicine Graduate School of Medical Science Kyoto Prefectural University of Medicine Kyoto Japan; 3 Department of Cardiovascular Medicine Japanese Red Cross Kyoto Daini Hospital Kyoto Japan

**Keywords:** persistent atrial fibrillation, atrial fibrillation duration, 12-lead electrocardiogram, machine learning, support system

## Abstract

**Background:**

Atrial fibrillation (AF) is a progressive disease, and its clinical type is classified according to the AF duration: paroxysmal AF, persistent AF (PeAF; AF duration of less than 1 year), and long-standing persistent AF (AF duration of more than 1 year). When considering the indication for catheter ablation, having a long AF duration is considered a risk factor for recurrence, and therefore, the duration of AF is an important factor in determining the treatment strategy for PeAF.

**Objective:**

This study aims to improve the accuracy of the cardiologists’ diagnosis of the AF duration, and the steps to achieve this goal are to develop a predictive model of the AF duration and validate the support performance of the prediction model.

**Methods:**

The study included 272 patients with PeAF (aged 20-90 years), with data obtained between January 1, 2015, and December 31, 2023. Of those, 189 (69.5%) were included in the study, excluding 83 (30.5%) who met the exclusion criteria. Of the 189 patients included, 145 (76.7%) were used as training data to build the machine learning (ML) model and 44 (23.3%) were used as test data for predictive ability of the ML model. Using a questionnaire, 10 cardiologists (group A) evaluated whether the test data (44 patients) included AF of more than a 1-year duration (phase 1). Next, the same questionnaire was performed again after providing the ML model’s answer (phase 2). Subsequently, another 10 cardiologists (group B) were shown the test results of group A, were made aware of the limitations of their own diagnostic abilities, and were then administered the same 2-stage test as group A.

**Results:**

The prediction results with the ML model using the test data provided 81.8% accuracy (72% sensitivity and 89% specificity). The mean percentage of correct answers in group A was 63.9% (SD 9.6%) for phase 1 and improved to 71.6% (SD 9.3%) for phase 2 (*P*=.01). The mean percentage of correct answers in group B was 59.8% (SD 5.3%) for phase 1 and improved to 68.2% (SD 5.9%) for phase 2 (*P*=.007). The mean percentage of answers that differed from the ML model’s prediction for phase 2 (percentage of answers where cardiologists did not trust the ML model and believed their own determination) was 17.3% (SD 10.3%) in group A and 20.9% (SD 5%) in group B and was not significantly different (*P*=.85).

**Conclusions:**

ML models predicting AF duration improved the diagnostic ability of cardiologists. However, cardiologists did not entirely rely on the ML model’s prediction, even if they were aware of their diagnostic capability limitations.

## Introduction

Atrial fibrillation (AF) is a progressive condition, with many cases starting as paroxysmal AF (PAF) and progressing to persistent AF (PeAF) and long-standing persistent AF (LsPeAF). PeAF is classified as AF lasting for less than 1 year, while LsPeAF is classified as AF lasting for more than 1 year [1,2]. Catheter ablation (CA) is a beneficial treatment for patients with AF, but the outcomes for LsPeAF are not as good as those for PAF or PeAF, as atrial degeneration becomes advanced due to long-term AF persistence [3]. Therefore, in the 2017 Heart Rhythm Society/European Heart Rhythm Association/European Cardiac Arrhythmia Society statement, the recommendation for CA for symptomatic LsPeAF is lower than that for PAF and PeAF [4]. The 2023 American College of Cardiology/American Heart Association/American College of Clinical Pharmacy/Heart Rhythm Society guidelines also state that CA is particularly effective in patients with AF of less than a 1-year duration [5], and the 2020 European Society of Cardiology guidelines list a longer duration of AF as a risk factor for recurrence when considering the indication for CA [6]. In addition, pulmonary vein isolation alone is often insufficient in cases with advanced atrial degeneration, as the involvement of maintenance mechanisms as well as triggering of AF is greater. In cases with LsPeAF, additional treatments other than pulmonary vein isolation such as linear ablation [7,8], complex fractionated atrial electrogram ablation [9], driver ablation [10,11], and low voltage area ablation [12] may also be considered. Estimation of the duration is therefore an important factor in determining the treatment strategy. Although the AF duration is an important finding for clinicians when deciding on a treatment strategy for AF, we often experience patients whose AF duration is unknown from the patient interview. In such cases, the cardiologists judge the AF duration based on clinical findings, such as an advanced age, a history of hypertension or heart failure, and the presence or absence of left atrial enlargement [13]. Thus, the objective of this study was to “improve the accuracy of the cardiologists’ diagnosis of the AF duration,” and the steps to achieve this objective were to “develop an AF duration prediction model” and to “validate the support performance of the prediction model.”

A small number of machine learning (ML) models have been developed to estimate the AF duration using only the characteristics of the fibrillation wave (*f* wave) on the 12-lead electrocardiogram (ECG) during AF. For example, the area under the curve (AUC) for a prediction using the amplitude of lead II was 0.77 and the AUC for a prediction using the dominant frequency (DF) in lead V1 was 0.63 [14]; a study reporting the prediction of the AF duration with *f* wave training using an ML model also had an AUC of 0.78 [15]. Therefore, in this study, we trained the ML prediction model based on multimodal data, including the ECG *f* wave characteristics, patient background, echocardiographic findings, and laboratory data.

## Methods

### Study Design and Population

The study included 272 patients with PeAF (aged 20-90 years) who were admitted for an initial CA at the Japanese Red Cross Kyoto Daini Hospital between January 1, 2015, and December 31, 2023, with available ECGs and patient background, echocardiography, and laboratory data within 3 months. A total of 189 (69.5%) patients were included in the study, excluding 14 (5.1%) patients whose ECG recorded a pacemaker rhythm, 30 (11%) whose QRS waves were difficult to exclude or whose extracted *f* waves were too short for a feature analysis, and 39 (14.3%) whose AF duration was difficult to determine.

The following methods were used to define PeAF (AF duration of less than 1 year) and LsPeAF (AF duration of 1 year or more). First, PeAF was defined if any of the following conditions were met: (1) sinus rhythm was recorded on the ECG within 1 year, excluding postdefibrillation ECGs, followed by a consecutive recording of AF; and (2) the onset of clinical symptoms or an irregular pulse clearly related to AF was confirmed within 1 year, followed by a consecutive ECG recording of AF. Second, LsPeAF was defined as any of the following: (1) patients with ECG recordings of AF confirmed for more than 1 year consecutively and (2) patients with onset of clinical symptoms or an irregular pulse clearly related to AF more than 1 year prior, followed by consecutive ECG recordings of AF within 1 year. Cases that did not meet any of the above conditions were excluded as cases with an unknown duration.

**Figure 1:**
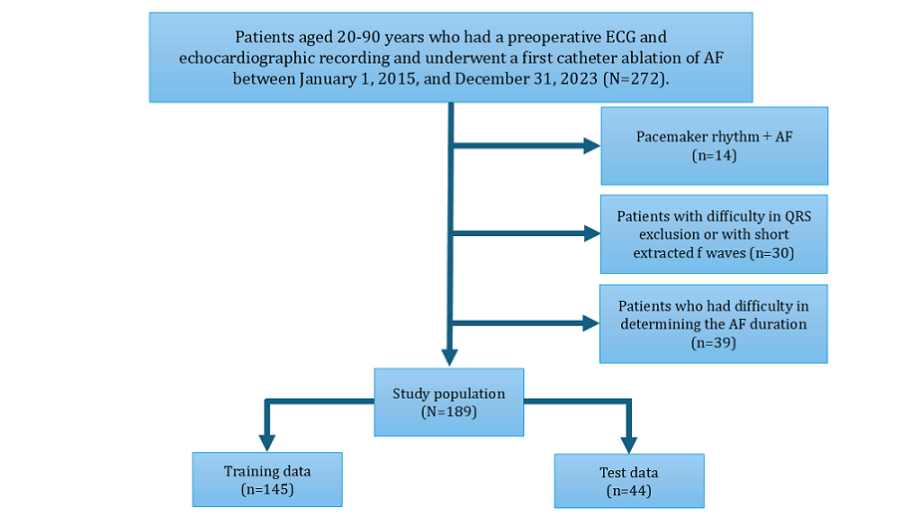
Study design and population. AF: atrial fibrillation; ECG: electrocardiogram.

The ML model was developed and cross-validated to determine whether the duration of AF was more than 1 year, using 145 patients with a registration period from January 1, 2015, to March 31, 2023, as training data. The predictive ability of the ML model was tested using 44 patients whose registration period was from April 1, 2023, to December 31, 2023, as test data (Figure 1).

**Figure 2:**
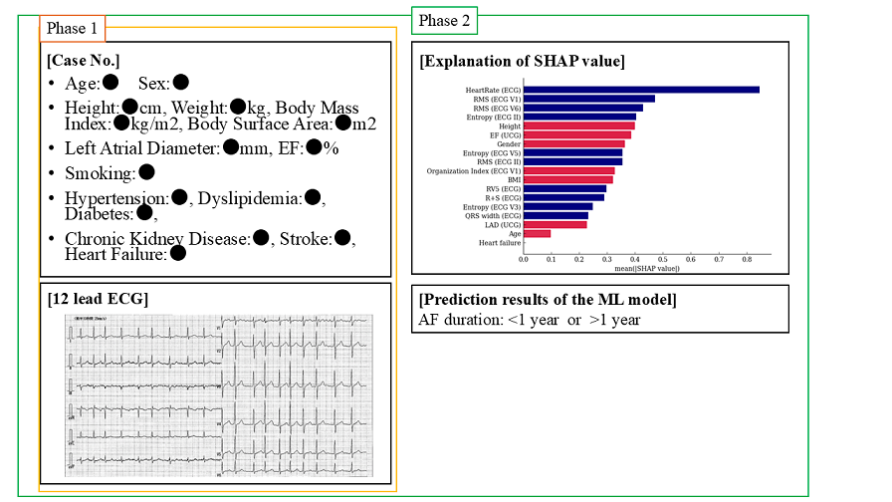
Case presentation during phases 1 and 2. In phase 1, the cardiologist made a decision on the AF duration by presenting the information and ECG of only 1 case. In phase 2, in addition, the decision was made by presenting the SHAP values and predicted results of the ML model. AF: atrial fibrillation; ECG: electrocardiogram; EF: ejection fraction; LAD: left atrial diameter; ML: machine learning; RMS: root mean square; SHAP: Shapley additive explanation; UCG: ultrasound cardiography.

In phase 1, a total of 10 cardiologists (group A) evaluated the test data of 44 patients using their ECGs and patient information and answered whether each patient had an AF of at least a 1-year duration. In phase 2, they answered that again after being presented with the predictive results of the ML model and the Shapley additive explanation (SHAP) values, which calculated the contribution of each variable feature to the predictive results of the model. The case presentation in phases 1 and 2 is shown in Figure 2. Following that, group B was shown with the diagnostic results of group A, and the same 2-phase tests were conducted as in group A, with a prior recognition of the cardiologists’ ability to diagnose the AF duration. Finally, all cardiologists were asked whether they attached any importance to each of the characteristics as a decision-making factor.

### Data Analysis

#### Feature Analysis From the 12-Lead ECG Data

The heart rate (HR), QRS width, S1 amplitude, RV5 amplitude, and R+S (=S1 amplitude+RV5 amplitude) were referenced from 10-second ECG data analyzed using a 12-lead ECG recording device (FCP-8800 or FCP-8700; Fukuda Denshi Co, Ltd).

#### F Wave Extraction

**Figure 3:**
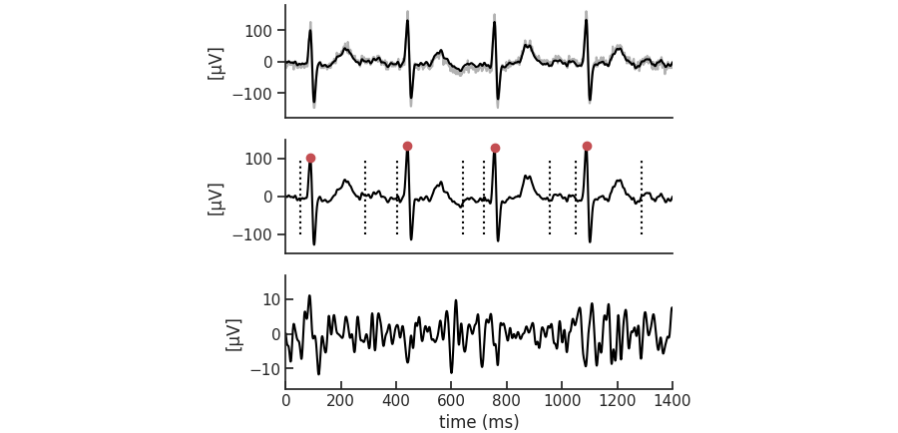
F wave extraction. (A) The R wave time was peak detected. (B) The Q wave time was obtained by subtracting 37 ms, the typical ventricular activation time, from the R wave time; the T wave time was obtained by adding 200 ms to the R wave time. (C) For each QRST interval, the f wave was extracted using a principal component analysis.

The *f* wave was extracted from 10-second ECG data. Bandpass filtering was used to reduce any baseline meandering and high-frequency noise. The *f* waves were in the 4-9 Hz bandwidth, so the cutoff frequency was set to 0.8-40 Hz. The QRST interval was detected for QRST cancellation; the *R* wave time was peak-detected by applying the Pan-Tompkins algorithm. The *Q* wave time was obtained by subtracting 37 ms, the typical ventricular activation time, from the *R* wave time; the *T* wave time was obtained by adding 200 ms to the *R* wave time. For each QRST interval, the *f* wave was extracted using a principal component analysis. To suppress any residual QRST features, filtering was performed by a bandpass filter. The *f* waves were in the 4-9 Hz bandwidth, so the cutoff frequency was set to 3-20 Hz (Figure 3).

#### F Wave Features

The root mean square (RMS) of the amplitude was defined as the square root of the mean square, which is the arithmetic mean of the squares of the signal amplitudes in the time domain. Sample entropy (SampEn) was used in the evaluation of *f* wave irregularities. The SampEn is a measure of entropy first proposed by Richman and Moorman [16], a method designed to reduce the bias of approximate entropy and to obtain more robust statistics. The SampEn was defined by the following equation:



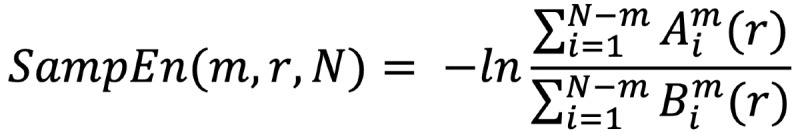



where 
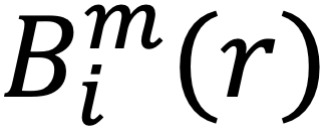
 is *(N – m)*^–1^ times the number of 
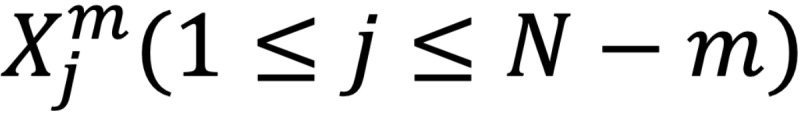
 that meets 
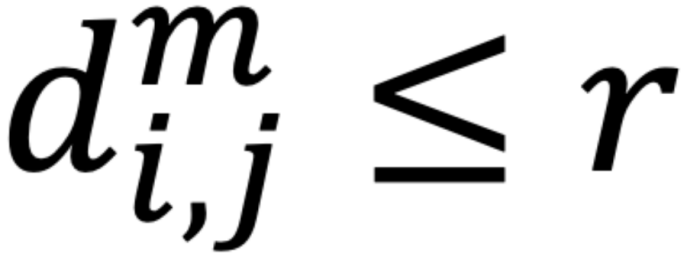
 and 
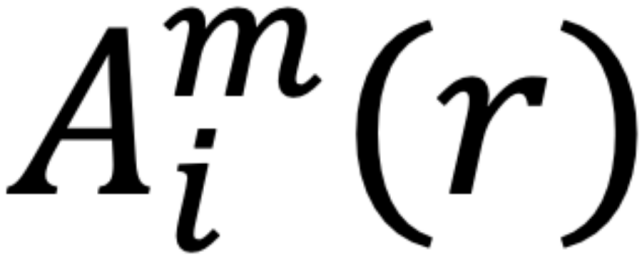
 is *(N – m)*^–1^ times the number of 
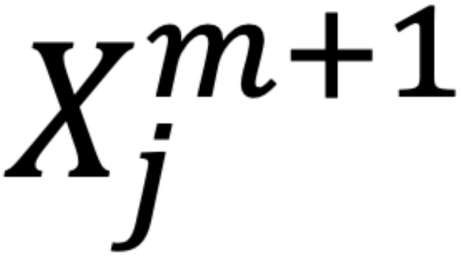
 that meets 
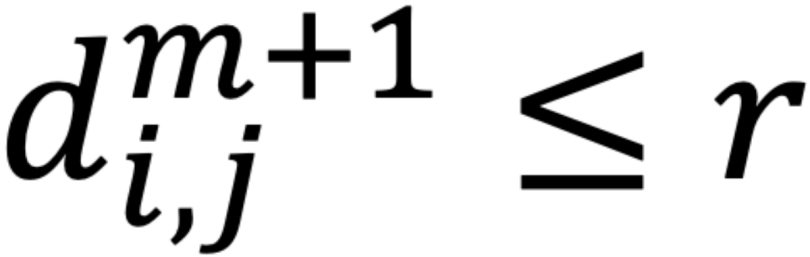
 for all 1 ≤ *j* ≤ *N* – *m*.

A threshold value of 3.5 and a sample size of 3 were set. After a fast Fourier transform, the DF and organization index (OI) were determined. The DF is one of the most widely used indices for the frequency analysis of *f* waves, and the frequency with the highest power value in the frequency distribution is defined as the DF. The OI was used as a measure of *f* wave organization. The OI was defined as the ratio of the area under the highest peak and its harmonics (not including the fifth harmonic peak) to the rest of the spectrum in the 3-15 Hz bandwidth.

### Statistical Analysis

Continuous variables are presented as the mean (SD). Categorical variables are presented as frequencies (percentages). Statistical significance was determined using the Mann-Whitney *U* test for continuous variables and the chi-square test for categorical variables; a *P* value <.05 was considered statistically significant. Python software (version 3.10.12; Python Software Foundation) was used for statistical analysis.

The percentage of correct answers for each cardiologist was calculated from the results of the cardiologist’s test and compared between phase 1 and phase 2 in group A and group B, respectively, using a paired sample 2-tailed *t* test.

#### ML Model and Cross-Validation

The model to predict long-term PeAF was built based on an ML algorithm. The objective variables were PeAF (duration of less than 1 year) and LsPeAF (duration of more than 1 year) defined earlier. The model construction was performed in the following steps: (1) step 1 involves the investigation of the ML algorithms; (2) step 2 involves feature selection; and (3) step 3 includes model training and evaluation.

#### Step 1

To select the appropriate ML algorithm for the research data, we used DataRobot to investigate more than 1000 different types of ML algorithms. The results of the survey showed that the gradient-boosting decision tree was highly accurate for the study dataset. Therefore, 2 ML algorithms, XGBoost (extreme gradient boosting) and LightGBM (a gradient-boosting decision tree model), were selected for the model development.

#### Step 2

In order to prevent overfitting with the ML algorithms, an appropriate number of feature selections was performed. To account for the effect of multicollinearity on the model, one of the features was excluded from the features with a correlation coefficient between the features greater than 0.7. The feature selection used a stepwise variable reduction method to search for the optimal combination of features. In particular, the *Boruta* package [17] was used to exclude any features that did not contribute to discriminating between PeAF and LsPeAF.

Four models were developed according to the features in order to evaluate the incremental benefits gained by adding each feature (Multimedia Appendix 1). Model 1 consisted of the age, sex, body size (height, weight, BMI, and body surface area), and past history. Model 2 further included the echocardiographic data (left atrial diameter [LAD], ejection fraction, and mitral regurgitation greater than moderate). Model 3 also included the ECG data (HR, QRS width, SV1 amplitude, RV5 amplitude, and R+S). Model 4 also included the *f* wave features (RMS, DF, entropy, and OI). Those modalities were designed according to common clinical measures.

#### Step 3

The dataset was divided into training data (145/189, 76.7%) and test data (44/189, 23.3%). The test data were set to be in the future in time relative to the training data. No test data were set for external validation. The training data were divided into 5 partitions and stratified sampling was performed to ensure an equal target distribution. The ML models were trained using Bayesian optimization methods with a nested 5-partition cross-validation.

For the prediction models selected from the aforementioned evaluations, the TreeExplainer of the SHAP method was used to estimate the extent to which each feature contributed to the decision-making of the prediction model. The feature importance was arranged in descending order based on SHAP values.

The cutoff points for calculating the other evaluation metrics—accuracy, *F*_1_-score, sensitivity, specificity, positive predictive value, and negative predictive value—were Youden indices computed from the training dataset.

### Ethical Considerations

The study was approved by the Medical Ethics Review Committee of the Kyoto Prefectural University of Medicine (ERB-C-2195-1). Informed consent was given on an opt-out basis, and all data were anonymized. The study was conducted without offering any financial or material rewards to the participants.

## Results

### Clinical Characteristics

Of the 189 patients studied, 130 (68.8%) had PeAF and 59 (31.2%) had LsPeAF (Table 1). There was no significant difference in age (*P*=.50), and in terms of sex, there were significantly more male patients in the LsPeAF group (*P*=.01). There were no significant differences in the medical history between the 2 groups (all *P*>.05). On examination, echocardiography showed no significant difference in LAD between the two groups (*P*=.11). The ECG data showed that the HR was significantly lower in the LsPeAF group (*P*<.001), but there were no significant differences in the QRS wave width or amplitude (all *P*>.05).

Among the *f* wave characteristics, the aVR and V1 RMS were significantly lower in the LsPeAF group (*P*=.05 and *P*=.04, respectively), and the other inductions tended to have lower mean values overall, but there were no significant differences (all *P*>.05). No significant differences were observed in the DF and OI in any of the inductions (all *P*>.05; Table 2).

**Table 1:** Baseline characteristics.

Characteristics		Total (N=189)	PeAF^a^ (n=130)	LsPeAF^b^ (n=59)	*P* value
**Age (years), mean (SD)**		71.36 (9.8)	70.95 (10.06)	72.25 (9.22)	.50
**Sex** **(male), n (%)**		133 (70.4)	84 (65)	49 (83.1)	.01
**Height (m), mean (SD)**		162.91 (9.51)	162.27 (10.21)	164.33 (7.63)	.25
**Weight (kg), mean (SD)**		64.84 (13.13)	64.24 (13.72)	66.14 (11.73)	.19
**BMI (kg/m^2^), mean (SD)**		24.31 (3.83)	24.27 (3.95)	24.4 (3.59)	.95
**BSA^c^ (m^2^), mean (SD)**		1.69 (0.2)	1.68 (0.21)	1.72 (0.17)	.17
**Past history, n (%)**					
	Heart failure	66 (35)	46 (35.4)	20 (34)	.84
	Stroke	23 (12.2)	16 (12.3)	7 (12)	.93
	Hypertension	129 (68.3)	86 (66.2)	43 (73)	.36
	Diabetes	40 (21.2)	25 (19.2)	15 (25.4)	.33
	Dyslipidemia	91 (48.1)	58 (45)	33 (56)	.15
	CKD^d^	54 (29)	36 (28)	18 (30.5)	.69
	CPD^e^	7 (4)	6 (5)	1 (1.7)	.32
**Echocardiographic data**					
	LAD^f^, mean (SD)	44.49 (7.05)	44.05 (6.07)	45.47 (8.82)	.11
	Ejection fraction, mean (SD)	62.89 (10.22)	62.02 (11.29)	64.81 (7.03)	.25
	MR^g^>moderate, n (%)	17 (9)	14 (11)	3 (5.1)	.21
**ECG^h^ data, mean (SD)**					
	Heart rate	83.33 (18.37)	87.59 (18.03)	73.96 (15.51)	<.001
	QRS width	101.16 (14.52)	101.94 (15.97)	99.46 (10.58)	.62
	SV1 amplitude	0.72 (0.44)	0.75 (0.48)	0.64 (0.31)	.35
	RV5 amplitude	1.8 (0.68)	1.81 (0.71)	1.77 (0.63)	.96
	R＋S^i^	2.52 (0.86)	2.56 (0.89)	2.42 (0.78)	.46

^a^PeAF: persistent atrial fibrillation.

^b^LsPeAF: long-standing persistent atrial fibrillation.

^c^BSA: body surface area.

^d^CKD: chronic kidney disease.

^e^CPD: chronic pulmonary disease.

^f^LAD: left atrial diameter.

^g^MR: mitral regurgitation.

^h^ECG: electrocardiogram.

^i^R+S: SV1 amplitude+RV5 amplitude.

**Table 2:** *F* wave features.

*F* wave characteristics and leads			Total (N=189), mean (SD)		PeAF^a^ (n=130), mean (SD)		LsPeAF^b^ (n=59), mean (SD)		*P* value
**RMS^c^ (μV)**									
	I	8.56 (2.48)		8.72 (2.55)		8.21 (2.29)		.14	
	II	13.66 (4.67)		14.11 (4.89)		12.65 (4.03)		.07	
	aVR	9.23 (2.73)		9.49 (2.81)		8.66 (2.48)		.049	
	aVL	9.16 (2.59)		9.42 (2.73)		8.58 (2.18)		.08	
	V1	22.14 (8.91)		22.81 (9.01)		20.64 (8.56)		.04	
	V2	19.05 (7.91)		19.52 (8.64)		18.02 (5.91)		.49	
	V3	16.81 (7.07)		17.07 (7.47)		16.26 (6.15)		.36	
	V4	16.17 (5.84)		16.21 (5.37)		16.09 (6.8)		.44	
	V5	14.93 (5.08)		15.13 (4.99)		14.48 (5.29)		.21	
	V6	12.0 (3.64)		12.31 (3.76)		11.34 (3.31)		.06	
**Dominant frequency (Hz)**									
	I	6.23 (0.97)		6.19 (1.02)		6.33 (0.87)		.33	
	II	6.16 (0.93)		6.16 (0.93)		6.16 (0.94)		.98	
	aVR	6.22 (0.97)		6.12 (0.95)		6.44 (0.99)		.06	
	aVL	6.28 (0.91)		6.2 (0.93)		6.47 (0.85)		.06	
	V1	6.3 (0.95)		6.33 (0.93)		6.24 (0.99)		.36	
	V2	6.35 (0.93)		6.34 (0.91)		6.37 (0.99)		.97	
	V3	6.29 (1.01)		6.25 (0.96)		6.37 (1.11)		.52	
	V4	6.24 (1.05)		6.15 (0.97)		6.43 (1.18)		.16	
	V5	6.22 (1.1)		6.13 (1.07)		6.42 (1.14)		.11	
	V6	6.17 (1.12)		6.17 (1.1)		6.17 (1.16)		.92	
**Entropy**									
	I	0.94 (0.14)		0.95 (0.14)		0.93 (0.16)		.12	
	II	1.1 (0.17)		1.12 (0.16)		1.05 (0.17)		.01	
	aVR	0.94 (0.14)		0.96 (0.13)		0.91 (0.14)		.01	
	aVL	0.94 (0.12)		0.96 (0.12)		0.91 (0.12)		.01	
	V1	1.18 (0.18)		1.2 (0.18)		1.13 (0.18)		.01	
	V2	1.15 (0.17)		1.17 (0.17)		1.11 (0.15)		.045	
	V3	1.09 (0.15)		1.12 (0.16)		1.04 (0.13)		.003	
	V4	1.07 (0.14)		1.09 (0.15)		1.03 (0.12)		.01	
	V5	1.04 (0.14)		1.06 (0.14)		1.0 (0.13)		.01	
	V6	0.99 (0.14)		1.01 (0.14)		0.96 (0.13)		.02	
**Organization index**									
	I	0.27 (0.07)		0.27 (0.07)		0.27 (0.06)		.90	
	II	0.3 (0.08)		0.29 (0.08)		0.3 (0.08)		.54	
	aVR	0.28 (0.08)		0.28 (0.08)		0.27 (0.08)		.23	
	aVL	0.29 (0.08)		0.29 (0.08)		0.28 (0.08)		.37	
	V1	0.31 (0.09)		0.31 (0.09)		0.33 (0.08)		.14	
	V2	0.3 (0.08)		0.3 (0.08)		0.31 (0.09)		.98	
	V3	0.29 (0.08)		0.29 (0.07)		0.29 (0.08)		.98	
	V4	0.27 (0.07)		0.27 (0.07)		0.26 (0.07)		.37	
	V5	0.26 (0.07)		0.26 (0.08)		0.25 (0.07)		.23	
	V6	0.27 (0.07)		0.27 (0.07)		0.27 (0.07)		.87	

^a^PeAF: persistent atrial fibrillation.

^b^LsPeAF: long-standing persistent atrial fibrillation.

^c^RMS: root mean square

### Predictive Accuracy of the ML Models

**Figure 4:**
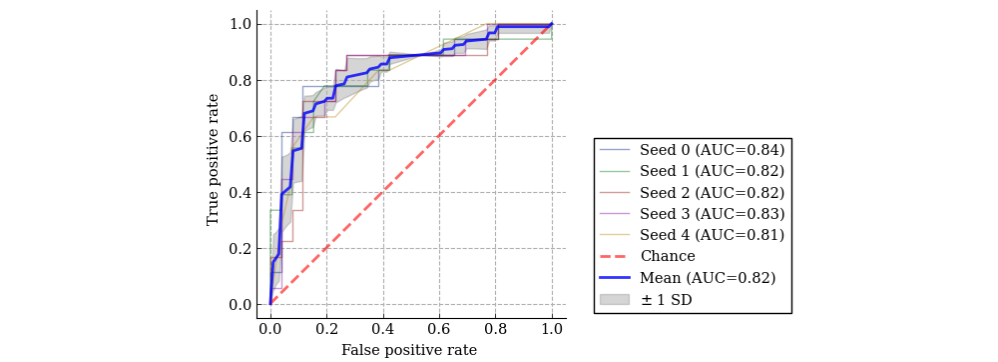
Predictive accuracy of the ML model using ROC curves. A random seed was set 5 times and the mean ROC-AUC was calculated. AUC: area under the curve; ML: machine learning; ROC: receiver operating characteristic.

In this study, model 4, trained with XGBoost, which had the highest AUC among the ML models created, was adopted (Multimedia Appendices 2 and 3). To evaluate the prediction accuracy of the ML model, a random seed was set 5 times, and the mean receiver operating characteristic–AUC curve was calculated; the mean AUC of those 5 was 0.82 (Figure 4).

**Figure 5:**
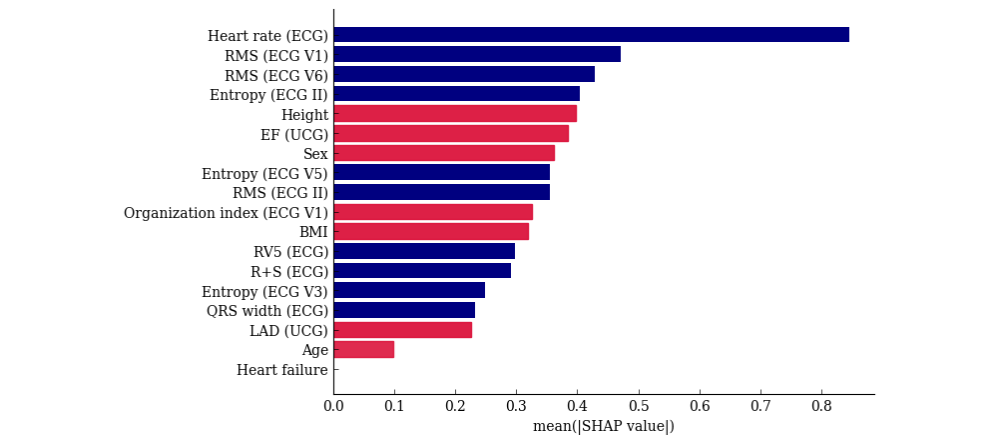
SHAP value. The red bars indicate a positive correlation and the blue bars a negative correlation. ECG: electrocardiogram; EF: ejection fraction; LAD: left atrial diameter; RMS: root mean square; SHAP: Shapley additive explanation; UCG: ultrasound cardiography.

In the SHAP value, the ML model gave the highest importance to HR. The following weightings were then assigned in the following order: RMS, SampEn, amplitude, left ventricular ejection fraction, sex, OI, BMI, QRS wave amplitude, QRS width, LAD, and age (Figure 5).

The predicted results for the test data were 0.82 for accuracy, 0.72 for sensitivity, 0.81 for specificity, 0.81 for positive predictive value, 0.82 for negative predictive value, and 0.77 for *F*_1_-score.

### Cardiologists’ Decisions With and Without ML Model Support

**Figure 6:**
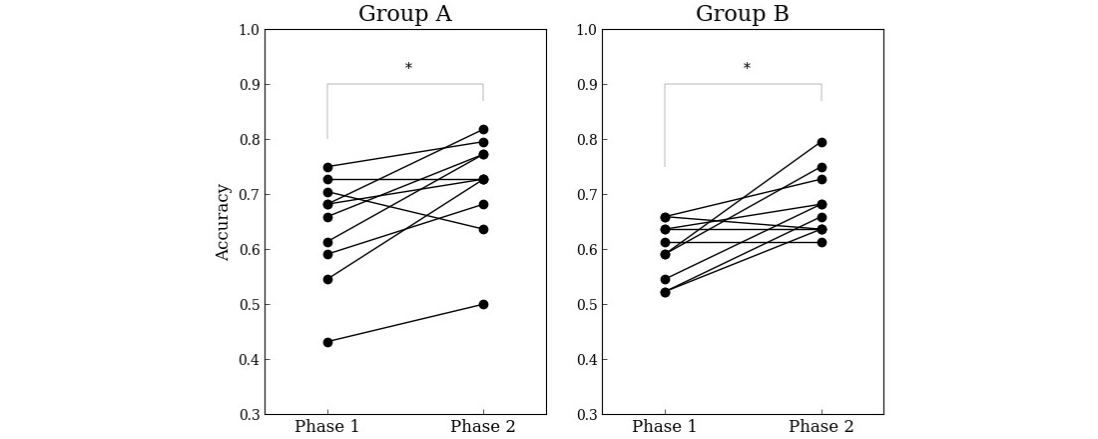
Improvement in the prediction accuracy by cardiologists after being assisted by the machine learning model. The dots connected by lines indicate the results for the same cardiologist. The asterisks mean statistically significant differences. *P<.05.

The diagnostic accuracy of the AF duration by cardiologists is shown in Figure 6. The mean percentage of correct answers in group A was 63.9% (SD 9.6%) for phase 1 and improved to 71.6% (SD 9.3%) for phase 2 (*P*=.01). The mean percentage of correct responses in group B was 59.8% (SD 5.3%) for phase 1 and improved to 68.2% (SD 5.9%) for phase 2 (*P*=.007). There was no difference in the percentage of correct answers between groups A and B for phase 2 (*P*=.48). The diagnostic accuracy with the cardiologists significantly improved with ML model support in both groups. The mean percentage of answers that differed from the predictions of the ML model for phase 2 (percentage of answers where cardiologists did not trust the ML model and believed their own determination) was 17.3% (SD 10.3%) in group A and 20.9% (SD 5%) in group B, and there was no significant difference between groups A and B (*P*=.85).

### Posttest Questionnaire for Cardiologists

When a posttest questionnaire was conducted to find out which characteristics were important to the 20 cardiologists, 18 (90%) said that the LAD was important. This was followed by 17 (85%) cardiologists selecting the *f* wave amplitude in the 12-lead ECG, 16 (80%) selecting age, and 15 (75%) selecting the HR in the 12-lead ECG (Table 3).

**Table 3:** Results of a posttest questionnaire for cardiologists. The items are listed in descending order of importance for deciding the duration of atrial fibrillation.

Items	Respondents (N=20), n (%)
LAD^a^ (UCG^b^)	18 (90)
RMS^c^ mean (ECG^d^)	17 (85)
Age	16 (80)
Heart rate (ECG)	15 (75)
Heart failure	10 (50)
RMS SD (ECG)	8 (40)
EF^e^ (UCG)	7 (35)
BMI	7 (35)
Weight	5 (25)
QRS (ECG)	4 (20)
Sex	3 (15)
Hypertension	2 (10)
Height	1 (5)
Stroke	1 (5)
Smoker	0 (0)
Diabetes	0 (0)
Lipid	0 (0)

^a^LAD: left atrial diameter.

^b^UCG: ultrasound cardiography.

^c^RMS: root mean square.

^d^ECG: electrocardiogram.

^e^EF: ejection fraction.

## Discussion

### Principal Findings

Regarding the prediction of AF duration, the goal was to achieve a more accurate prediction by using ML models. As a result, the part that had previously relied on the cardiologists’ experience and subjectivity was supplemented, and the uncertainty related to the prediction was reduced. Next, we adopted an approach that combined the cardiologists’ diagnostic capability with the ML models. This enabled the objective prediction with the ML model to be referenced against the cardiologists’ experience and subjective judgment, thereby improving the overall quality of the cardiologists’ diagnostic process.

### Performance of the ML Model and the Cardiologists’ Reaction

There have been few reports that have validated the prediction of the duration using *f* wave characteristics: the AUC for the prediction using the amplitude of the *f* wave in lead II of the 12-lead ECG was 0.77, the AUC for the prediction using the DF of the *f* wave in lead V1 was 0.63 [14], and the AUC for the prediction using *f* wave learning with the ML model was 0.78 [15]. The ML model developed in this study was trained not only with the ECG characteristics including the *f* wave but also a past history and echocardiographic and laboratory data, which could improve the prediction accuracy to AUC 0.82 despite the small dataset. A validation using the test data also confirmed the result of an accuracy of 0.818. Therefore, in both groups A and B, the support of the ML model improved the ability of the cardiologists to diagnose the AF duration.

On the other hand, group A had a different answer selected for 24.3% of the ECGs compared to the ML model. One possible reason for that was likely to be that they believed their own decision over the ML support, as they did not know the superiority or inferiority of the cardiologists and ML model in predicting the duration of AF.

Therefore, an additional test was conducted with group B. The accuracy of the cardiologists’ diagnosis (percentage of correct answers in group A) was shown to group B in advance so that they were aware of the superiority or inferiority between the accuracy of the cardiologists’ diagnosis and the accuracy of the ML model. The same diagnostic test was then conducted with group B as in group A. Surprisingly, the percentage of cases selected that differed from the prediction of the ML model (21%) did not improve. That may have been due to the feature set of the model.

Among the SHAP values, the HR was the most correlated with the AF duration. Those facts are empirically recognized by cardiologists. In fact, 75% (15/20) of cardiologists consider HR to be important. For example, animal studies have shown that significant functional electrophysiological remodeling of the atrioventricular node occurs after prolonged AF, resulting in a spontaneous decrease in the ventricular HR [18]. Aging has also been shown to correlate with the atrioventricular node dysfunction as a result of atrioventricular node remodeling and ion channel expression [19]. In addition, patients with LsPeAF are often treated with drugs that control the HR, such as beta-blockers, calcium channel blockers, and digitalis. Those drugs decrease the HR and prevent it from becoming excessively fast. Taking those drugs over a long period of time may decrease the HR due to their cumulative effect. The dataset used in this study consisted of patients with AF scheduled for CA, and patients with a particularly large LAD tended to be excluded from the dataset at the discretion of their cardiologists because of their high risk of recurrence after CA. That selection bias may have been responsible for the discrepancy between the LAD characteristics and the ML prediction and cardiologist decision-making. The LAD can also be magnified by factors other than left atrial remodeling due to AF; Tsang et al [20] reported that left atrial enlargement is associated with congestive heart failure, vascular disease, transient ischemic attacks or strokes, and a history of smoking and is particularly strongly associated with diastolic dysfunction. Even in patients without a history of atrial arrhythmias or valvular disease, left atrial enlargement has been shown to increase with the severity of diastolic dysfunction, and remodeling due to AF (ie, LsPeAF) is not necessarily correlated with left atrial enlargement.

### Challenges for the Clinical Application of ML Models for Clinical Assistance

The lack of a difference in the diagnostic improvement between groups A and B indicated that cardiologists tend to trust their own experience when there is a discrepancy between their perception and the results of the ML model. That may be the case unless, for example, their judgment was clearly erroneous, in which case simply recognizing that the accuracy of the ML model was higher than the cardiologist’s diagnostic accuracy may not have been sufficient to influence their decision-making. Lee [21] noted that artificial intelligence (AI) and human predictive performance may differ depending on the type of patient, and a deeper understanding of AI and human predictive capabilities, respectively, will increase human reliance on AI. Therefore, for clinicians to trust AI, understanding why the algorithm came to the conclusion it did is important for them to trust AI. In this regard, in addition to the diagnostic explainability, one should also consider expressing predictions as probabilities. That is because greater clarity about the uncertainty of predictions may help cardiologists assess the risk and reliability of a diagnosis. For example, according to a study by Heuer and Breiter [22], users of ML models value not only the accuracy of the prediction but also the probability representation of the prediction. In other words, they may need a process that allows them to decide whether to perform additional tests or treatments based on the reasons for the prediction (visual presentation of the impact of each factor) and its probability.

### Limitations

This study had some limitations. First, the quality of the echo and ECG data and the problem of missing data may have affected the prediction accuracy of the ML model. Therefore, data cleaning and filtering were performed to exclude low-quality data and proceed with the analysis. As a result, the model’s prediction accuracy was 81.8%, with a sensitivity of 72% and specificity of 89%, thus confirming a certain degree of robustness. Second, this was a retrospective study and there may be some patients for whom the determination of the AF duration was uncertain. The determination of whether the duration was more than 1 year was based not only on interviews with the patients but also on physical examinations by cardiologists and ECG recordings. Furthermore, unclear symptoms and cases in which the timing was difficult to determine were excluded from this study, so we believe that the reliability and accuracy of the duration determination were high. Third, the study was not validated at other sites and the generalizability of the results to other sites and different populations was not confirmed. Also, the relatively small size of the dataset and the possibility of overfitting may have limited the robustness and generalizability of the model. Therefore, overfitting was avoided by “removing as many irrelevant variables as possible,” “cross-validation,” “validation with multiple seed values,” and “validation with test data from a different time period than the training data.” Those results confirmed that, even with the current dataset, the ML model complemented the cardiologists’ diagnostic ability and provided effective assistance in predicting the duration. However, further validation of the robustness and generalizability of the model is needed through prospective and larger datasets. Fourth, predicting the clinical outcomes of CA requires the unification of the CA procedures, which can only be adequately validated in prospective studies. Due to the limitations of the retrospective data in this study, we determined that it was not appropriate to predict the clinical outcomes, but in the future, we will evaluate the predictions regarding clinical outcomes using prospective data with a uniform CA technique. Lastly, the effect of rate control drugs as a confounding factor may have affected the HR. However, in this study, we dared to use data reflecting the actual clinical environment to improve the applicability of the model. In clinical practice, rate control with drugs is common, and by training the data to reflect that situation, we built a more practical model.

### Conclusions

An ML model was developed to diagnose the duration of AF. The diagnostic accuracy of the cardiologist was improved with the support of the ML model. However, cardiologists did not entirely rely on the prediction of the ML model, even if they were aware of the limitations of their diagnostic capability.

## Appendix

Multimedia Appendix 1 The feature selection results for each machine learning method and feature modality.

Multimedia Appendix 2 The ROC-AUC of the prediction models for each machine learning method and feature modality. AUC: area under the curve; ROC: receiver operating characteristic.

Multimedia Appendix 3 The ROC curves of the prediction models for each machine learning method and feature modality. ROC: receiver operating characteristic.

## Abbreviations

AF: atrial fibrillation
AI: artificial intelligence
AUC: area under the curve
CA: catheter ablation
DF: dominant frequency
ECG: electrocardiogram
HR: heart rate
LAD: left atrial diameter
LsPeAF: long-standing persistent atrial fibrillation
ML: machine learning
OI: organization index
PAF: paroxysmal atrial fibrillation
PeAF: persistent atrial fibrillation
RMS: root mean square
SampEn: sample entropy
SHAP: Shapley additive explanation
XGBoost: extreme gradient boosting
