# Supplementary material for: Using Machine Learning to Predict the Duration of Atrial Fibrillation: Model Development and Validation
Source: JMIR Med Inform. 2024 Nov 22;12:e63795. doi: 10.2196/63795 (PMC11624443; doi:10.2196/63795)
Supplement: Multimedia Appendix 1 [file medinform_v12i1e63795_app1.docx]

**Appendix1:** The feature selection results for each machine learning method and feature modality

| **Model Type** | | **XGBoost** | **LightGBM** |
| --- | --- | --- | --- |
| Model1 | Baseline | Age, Gender, Height, BMI^a^ | Age, Gender, Height, BMI^a^ |
|  | Past History | Heart Failure, Hypertension, Dyslipidemia, CKD^b^ | Heart Failure, Dyslipidemia, CKD^b^ |
| Model2 | Baseline | Age, Gender, Height, BMI^a^ | Age, Gender, Height, BMI^a^ |
|  | Past History | Heart Failure, Hypertension, Dyslipidemia, Diabetes, CKD^b^ | Heart Failure, Dyslipidemia, CKD^b^ |
|  | Echocardiographic Data | LAD^c^, Ejection Fraction | LAD^c^, Ejection Fraction |
| Model3 | Baseline | Age, Gender, Height, BMI^a^ | Age, Gender, Height, BMI^a^ |
|  | Past History | Heart Failure, Dyslipidemia | Heart Failure, Dyslipidemia, CKD^b^ |
|  | Echocardiographic Data | LAD^c^, Ejection Fraction | LAD^c^, Ejection Fraction |
|  | ECG Data | Heart Rate, QRS width, SV1 amplitude, RV5 amplitude, R+S^d^ | Heart Rate, QRS width, SV1, RV5 amplitude, R+S^d^ |
| Model4 | Baseline | Age, Gender, Height, BMI^a^ | Age, Gender, Height, BMI^a^ |
|  | Past History | Heart Failure | Heart Failure, Dyslipidemia |
|  | Echocardiographic Data | LAD^c^, Ejection Fraction | LAD^c^, Ejection Fraction |
|  | ECG Data | Heart Rate, QRS width, RV5 amplitude, R+S^d^ | Heart Rate, SV1, RV5 amplitude, R+S^d^ |
|  | F-wave features | RMS^e^_II, RMS^e^_V1, RMS^e^_V6, EN^f^_II, EN^f^_V3, EN^f^_V5, OI^g^_V1 | RMS^e^_II, RMS^e^_V1, RMS^e^_V6, EN^f^_II, EN^f^_V3, EN^f^_V5, OI^g^_V1 |

^a^BMI: Body Mass Index

^b^CKD: Chronic Kidney Disease

^c^LAD: Left Atrial Diameter

^d^R+S: SV1 amplitude+RV5 amplitude

^e^RMS: Root Mean Square

^f^EN: Entropy

^g^OI: Organization Index
