# Supplementary material for: Using Machine Learning to Predict the Duration of Atrial Fibrillation: Model Development and Validation
Source: JMIR Med Inform. 2024 Nov 22;12:e63795. doi: 10.2196/63795 (PMC11624443; doi:10.2196/63795)
Supplement: Multimedia Appendix 2 [file medinform_v12i1e63795_app2.docx]

**Appendix2:** The ROC-AUC of the prediction models for each machine learning method and feature modality

| **Model Type** | **Model1** | **Model2** | **Model3** | **Model4** |
| --- | --- | --- | --- | --- |
| **XGBoost** | 0.669 (0.011) | 0.714 (0.013) | 0.761 (0.017) | 0.824 (0.009) |
| **LightGBM** | 0.693 (0.011) | 0.742 (0.011) | 0.779 (0.011) | 0.817 (0.013) |

Model1: Baseline and Past History

Model2: Baseline, Past History and Echocardiographic Data

Model3: Baseline, Past History, Echocardiographic Data and ECG Data

Model4: Baseline, Past History, Echocardiographic Data, ECG Data and F-wave features

Each model was trained with five different random seed values. The predictive ability of the models was evaluated by calculating the area under the ROC curve (ROC-AUC), and mean and standard deviation of five different random seeds were shown in the Table.

ECG: Electrocardiogram, ROC: Receiver Operating Characteristic, AUC: Area Under the Curve
