# Supplementary material for: Using Machine Learning to Predict the Duration of Atrial Fibrillation: Model Development and Validation
Source: JMIR Med Inform. 2024 Nov 22;12:e63795. doi: 10.2196/63795 (PMC11624443; doi:10.2196/63795)
Supplement: Multimedia Appendix 3 [file medinform_v12i1e63795_app3.docx]

**Appendix3:** The ROC curves of the prediction models for each machine learning method and feature modality

|  | **XGBoost** | **LightGBM** |
| --- | --- | --- |
| **Model1** | 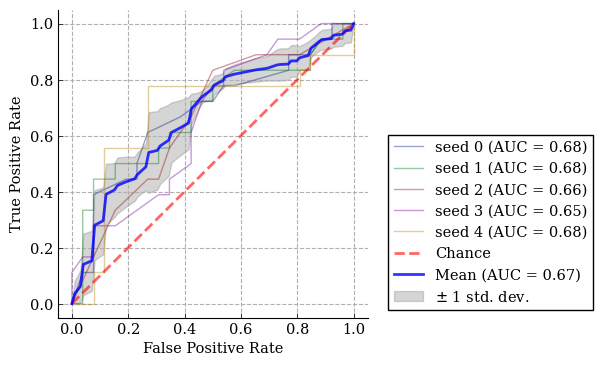 | 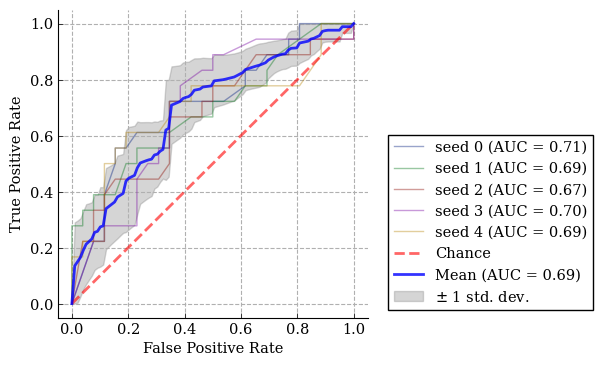 |
| **Model2** | 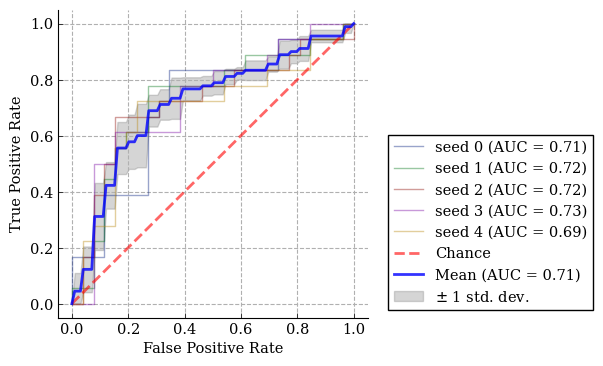 | 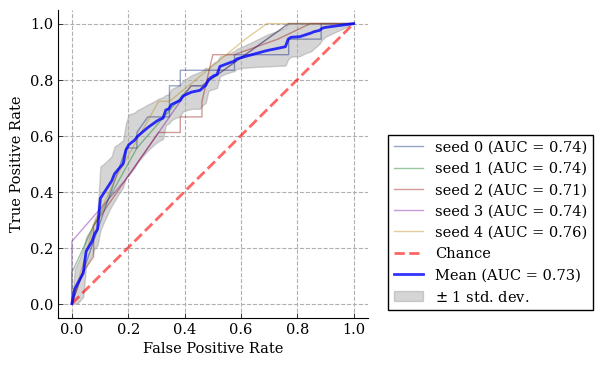 |
| **Model3** | 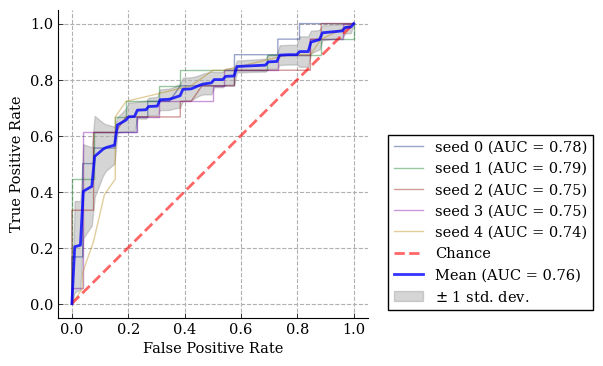 | 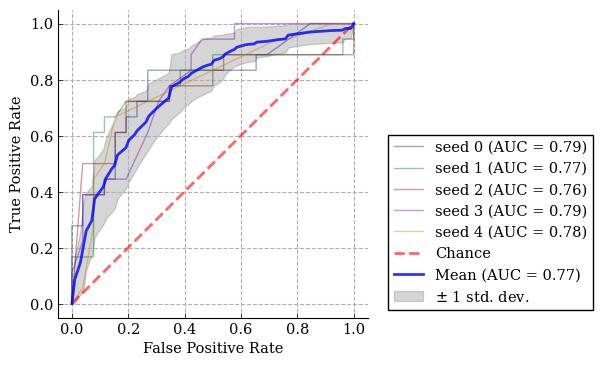 |
| **Model4** | 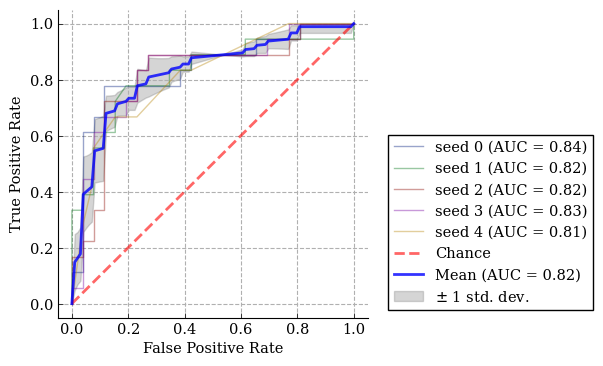 | 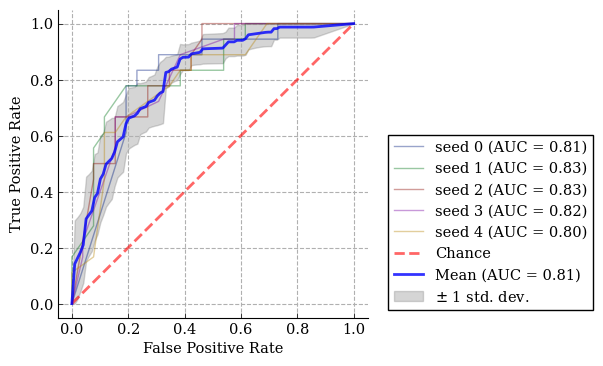 |

Model1: Baseline and Past History

Model2: Baseline, Past History and Echocardiographic Data

Model3: Baseline, Past History, Echocardiographic Data and ECG Data

Model4: Baseline, Past History, Echocardiographic Data, ECG Data and F-wave features

ROC: Receiver Operating Characteristic, AUC: Area Under the Curve, std dev: Standard Deviation, ECG: Electrocardiogram
